# A Biaxial Strain Sensor Using a Single MoS_2_ Grating

**DOI:** 10.1186/s11671-021-03493-3

**Published:** 2021-02-10

**Authors:** Junxiang Xiang, Wenhui Wang, Lantian Feng, Chao Feng, Meng Huang, Ping Liu, XiFeng Ren, Bin Xiang

**Affiliations:** 1 Hefei National Research Center for Physical Sciences at the Microscale, Department of Materials Science and Engineering, CAS Key Lab of Materials for Energy Conversion, University of Science and Technology of China, Hefei, 230026 Anhui China; 2 Research Laboratory for Quantum Materials, Singapore University of Technology and Design, Singapore, 487372 Singapore; 3 Key Laboratory of Quantum Information, University of Science and Technology of China, Chinese Academy of Sciences, Hefei, 230026 China; 4 Shandong Provincial Key Laboratory of Preparation and Measurement of Building Materials, University of Jinan, Jinan, 250022 China

**Keywords:** Biaxial strain, Grating, Reflectance, MoS_2_, First principles

## Abstract

In this paper, we report a new type of MoS_2_-based grating sensor for in-plane biaxial strain gauges with a precision limit of ~ 1‰. The MoS_2_ grating is numerically simulated with different biaxial strains up to 5%. Our first-principles calculations reveal that the strain sensitivity of the MoS_2_ reflectance spectrum can be considered an additional strain sensor integrated with the grating structure, enabling the mapping of in-plane biaxial strains. Our experimental studies on a prototype MoS_2_-grating sensor further confirm that a strain component perpendicular to the grating period can cause intensity peak shifts in the grating’s first-order diffraction patterns. This work opens a new path towards the sensing of in-plane biaxial strain within a single-grating device. Our new approach is applicable for other materials that have predictable reflectance response under biaxial strains and the capacity to form a two-dimensional single-crystal layer.

**Supplementary Information:**

The online version contains supplementary material available at 10.1186/s11671-021-03493-3.

## Introduction

Flexible electronics technology has received widespread attention from the academic and industrial communities, but the design and application of microscale and nanoscale flexible devices is challenging due to difficulties in dynamic displacement and deformation monitoring [1–5]. Most conventional strain detection methods based on resistance strain gauges require a miniaturized sensor array [4, 6, 7], which is hard to produce for flexible electronic applications. Optics-based two-dimensional (2D) strain detection techniques, such as speckle interferometry, are superior to those based on piezoresistivity because of their higher precision [8]. However, their image correlation measurement strategy is challenged by the requirements of complex image processing technology [8–10]. A reflection grating can provide a high resolution for the strain measurement but lacks the capability to detect 2D strain within a single device [11].

Over the past years, 2D materials have attracted tremendous research effort. Following the introduction of grapheme [12, 13], the family of 2D materials has been enlarged by many new members such as double atomic thin black phosphorus [14], triple atomic thin transition metal dichalcogenides [15], quadruple atomic thin group-III metal monochalcogenides [16], and other nonlayered 2D materials [17]. Many interesting properties have been found in these materials, keeping them under the spotlight of materials science [18–24].

The transition metal dichalcogenides exhibit outstanding optical and mechanical properties [25–27]. For instance, MoS_2_ can tolerate as much as 19.5% [26] of biaxial strain accompanied by its reflectance modulation [28], and WSe_2_ can show notable Berry curvature dipole as well as nonlinear Hall effect via strain engineering [29]. Incorporating the strain-sensitivity of a material’s reflectance spectrum into the function of the reflection grating device can be an efficient way to extend strain measurements to biaxial detection within a single device. However, there are no reports of the reflection gratings combined with strain-induced material reflectance modulation for 2D strain sensing applications.

Here, we propose a new type of in-plane biaxial strain sensing technique involving the strain-sensitivity of MoS_2_ reflectance in a reflection grating sensor. First-principles calculations reveal that biaxial strains can shift the peak of the intensity distribution in diffraction patterns of a MoS_2_-based grating device because the reflectance of MoS_2_ is sensitive to the strain-induced deformation. This nonlinear peak shift is well demonstrated by adding a second-order term to the uniaxial-strain linear equation, from which the strain component perpendicular to the grating period direction can be extracted with a precision limit of ~ 1‰. Our experimental studies on a prototype MoS_2_-grating device confirm that the strain perpendicular to the grating period can induce an intensity peak shift of the grating’s first-order diffraction pattern. Our research shows the possibility of one-shot, in-plane biaxial strain gauges with a single grating sensor.

## Methods

### Theoretical Calculations for MoS_2_ Flake

The MoS_2_ optical responses to the strain are all studied by first-principle calculations performed with the Vienna Ab-initio Simulation Package (VASP) [30]. All-electron projector augmented wave (PAW) potentials [31] were used for all calculations. Geometric relaxation and static calculations were carried out with the Perdew-Burke-Ernzerhof (PBE) generalized gradient approximation (GGA) method [32]. Spin–orbit coupling (SOC) [33] was also included in the static calculations. To determine the optical properties, quasiparticle energies were first obtained by the GW method derived from Green function theory [34]. The wave function obtained from the static calculations and the quasiparticle energy from the GW calculations were used to perform the Bethe–Salpeter equation (BSE) calculations [35] to predict the dielectric constant.

A bulk crystal model was used to represent the MoS_2_ flake (that the optical property differences between stacks greater than five layers is negligible [36]). In the optimization calculations, the energy cutoff was set to 400 eV, and a Monkhorst–Pack k-point set of 15*15*4 was used to sample the Brillouin zone. The lattice parameters were first optimized as a reference for later calculations of the strained MoS_2_ flake. The optimized lattice parameters were *a* = *b* = 3.18 Å and *c* = 13.87 Å. The geometry was relaxed until the energy converged to 10^–5^ eV. Adopting a technique from previous literature [37], an energy cutoff of 300 eV and *k*-point set of 6 × 6 × 2 were used in the optical calculations. The static energy converged to 10^–6^ eV in all calculations. The diffraction patterns were simulated based on the Helmholtz–Kirchhoff theorem [38]. More details are provided in the Additional file 1.

### Preparation of MoS_2_ Sample for Spectrum Measurement

The MoS_2_ thin film was mechanically exfoliated from a commercial MoS_2_ single crystal (SPI Supplies) and transferred to a polydimethylsiloxane (PDMS) substrate with tape. After the transfer, another layer of PDMS was fabricated on the flake and substrate to enhance adhesion.

### Preparation of MoS_2_ Grating on the Flexible Substrate

The MoS_2_ thin film was mechanically exfoliated from a commercial MoS_2_ single crystal (SPI Supplies) and transferred to a polydimethylsiloxane (PDMS) substrate with tape. To fabricate the grating device, the MoS_2_ flake was firstly patterned into a grating structure by electron beam lithography (EBL). Then the patterned sample was etched by oxygen plasma with a power of 20 W. Finally, we obtained the MoS_2_-based grating device by washing away the PMMA.

### MoS_2_ Device Measurements

**Fig. 1:**
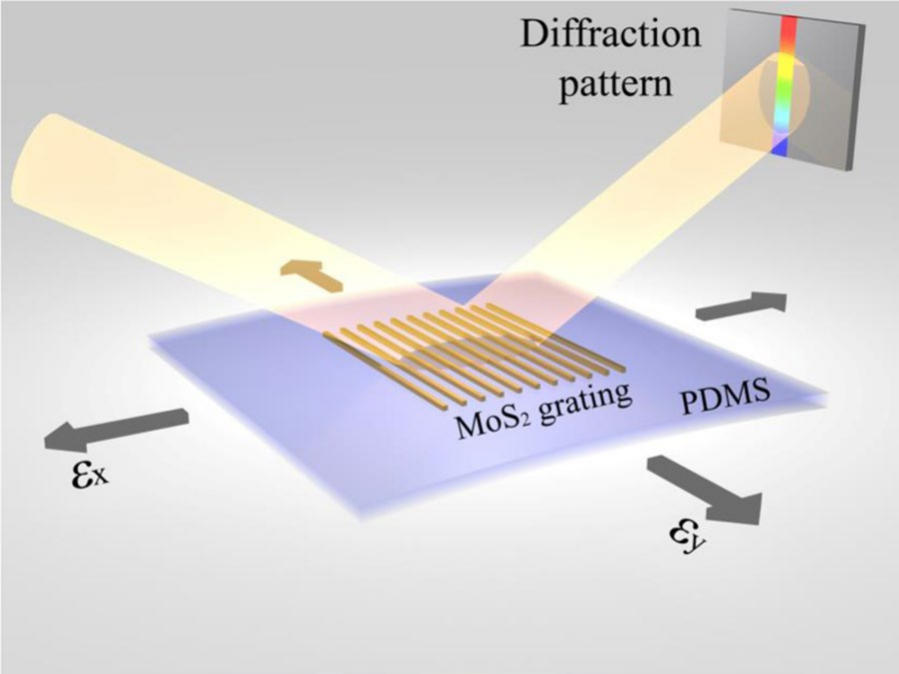
Schematic illustration of MoS_2_-based grating sensor on a flexible substrate of PDMS for biaxial strain gauges

A supercontinuum white light source (NKT Photonics SuperK Compact) is used as the excitation laser, which passes through one aperture and hits the flake sample or the grating sample at a certain angle relative to the sample plane, as shown in Fig. 1. In the reflectance measurement, the reflected laser is collected through an optic fiber connected to a spectrometer. The reflectance spectra under different strains are calculated from the data measured by the spectrometer. To test the MoS_2_ grating, the reflected laser is projected onto a white board and appears as a long elliptical light spot. Photos of the light spot are used to analyze the intensity distribution.

## Results and Discussion

In a conventional reflection grating sensor, a periodic structure of parallel grating strips can diffract the light, and the diffraction is utilized to measure a strain that is along the grating period direction by monitoring a location shift of the diffraction patterns [11]. Due to the periodic structure orientation, the strain sensing function of the reflection grating is limited to the in-plane uniaxial strain gauge (parallel to the periodic direction). To extend the reflection grating function for use in in-plane biaxial strain gauges, we propose that the intrinsic optical properties of the grating material, such as the strain-sensitivity of the material’s reflectance, can be considered as an additional strain sensor to detect in-plane strain components perpendicular to the periodic direction.

MoS_2_ has a layered structure: a layer of Mo atoms sandwiched between two layers of S atoms. The interaction between the layers is a weak van der Waals force. Here, we design a MoS_2_ flake-based reflection grating sensor (Fig. 1) and investigate the device diffraction patterns under different in-plane biaxial strains by first-principles calculations. The incident beam wavelength range in our calculation is from 400 to 850 nm. The diffraction grating can be described by:

1 $$d\left(\mathrm{sin}{\theta }_{i}-\mathrm{sin}{\theta }_{m}\right)=n\lambda$$

where $d$ is the distance between two adjacent grating strips, ${\theta }_{i}$ is the angle between the incident beam and the normal to the grating, ${\theta }_{m}$ is the angle between the diffraction beam and the normal when the diffraction beam has maxima, *n* is the diffraction order, and $\lambda$ is the beam wavelength [11]. From Eq. (1), we see that incident beams with different $\lambda$ must have different ${\theta }_{m}$. Therefore, a continuous wavelength beam causes a continuous series of diffraction spots corresponding to different ${\theta }_{m}$, forming an elliptical first-order diffraction pattern.

**Fig. 2:**
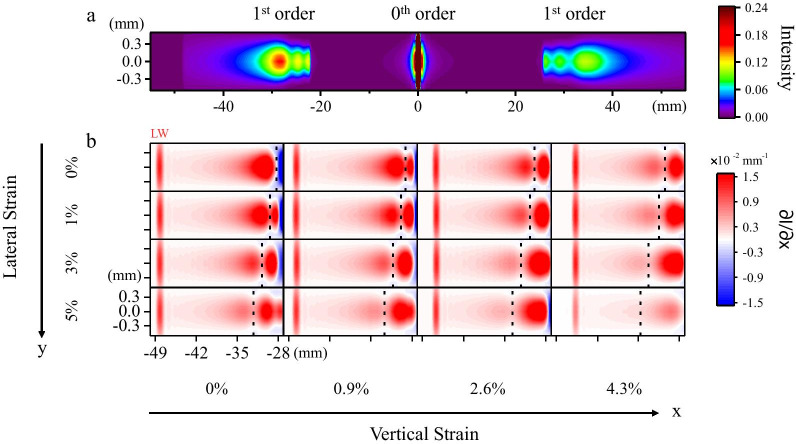
**a** Simulated image of the diffraction pattern. No strain was applied. The intensity is represented with colors. There is asymmetric behavior between the first-order diffraction spot on both sides of the zero-order beam because the screen in our simulation is set to be parallel to the grating instead of perpendicular to the reflection direction. **b** Simulated evolution of first-order diffraction spot under different biaxial strains. The intensity partial differential is represented with colors. The horizontal coordinate and vertical coordinate denote the position relative to the center of the zero-order diffraction spot. The peak is marked with a dashed line. From left to right, ${\varepsilon }_{x}$ was set as 0%, 0.9%, 2.6%, and 4.3%, respectively. From top to bottom, the ${\varepsilon }_{y}$ was 0%, 1%, 3%, and 5%, respectively

Figure 2a shows the simulated image of the diffraction patterns of the as-designed grating sensor with no strain applied. Figure 2b shows the intensity peak and pattern location evolution of the simulated first-order diffraction pattern of the device under different biaxial strains. The edge of the first-order diffraction pattern corresponding to the incident beam of 850 nm is labeled “LW”. When we apply an in-plane uniaxial tensile strain along the grating period direction (${\varepsilon }_{x}$), this strain can induce an increase in the spacing *d* between each strip. As a result, ${\theta }_{m}$ decreases because $d\mathrm{sin}{\theta }_{m}$ is constant for any given $\lambda$ and fixed ${\theta }_{i}$. Therefore, when we gradually increase the strain ${\varepsilon }_{x}$ from 0 to 4.3%, the location of each point in the first-order diffraction pattern moves closer to the center of the zero-order diffraction spot in a proportional relationship with the corresponding beam wavelength, which is consistent with the function of the conventional reflection grating sensor [11].

An incident beam with a longer wavelength $\lambda$ has a larger ${\theta }_{m}$ variation, so the LW edge has the most apparent location shift. However, when an in-plane tensile strain perpendicular to the grating period direction (${\varepsilon }_{y}$) is simultaneously applied, an intensity peak shift is observed within the first-order diffraction pattern, as marked by a dashed line in Fig. 2b. When the strain ${\varepsilon }_{y}$ increases from 0 to 5%, the intensity peak shifts further away from the center of the zero-order diffraction spot. We attribute this peak shift of the intensity distribution to the strain-induced modulation of the MoS_2_ reflectance. Previous literature has reported that the reflectance spectrum of MoS_2_ can be tuned by an external strain [28], and the reflectance is equal to the intensity ratio of the diffracted beam to the incident beam of the reflection grating. Therefore, the intensity of the beams with different wavelengths diffracted by the MoS_2_ grating can be modulated by the in-plane strains. Meanwhile, no LW edge location shift occurs because the strain ${\varepsilon }_{y}$ exerts no impact on the grating period.

**Fig. 3:**
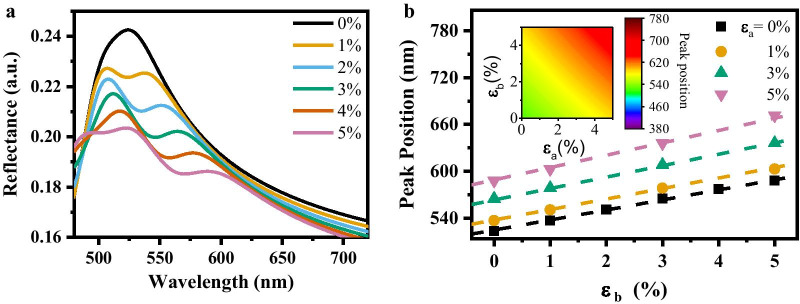
**a** Reflectance spectra of the MoS_2_ flake as a function of wavelength under different uniaxial strains. **b** Peak positions of the reflectance spectra of the MoS_2_ flake under different biaxial strains. Dashed lines represent the fitting curves. Inset: The mapping image of peak positions from the fitting equation

Figure 3a shows the linear behavior in the peak shifts of the MoS_2_ reflectance spectra when a uniaxial tensile strain along the lattice vector ${\varvec{b}}$ of MoS_2_ is applied. This uniaxial tensile strain causes a peak-position redshift in the MoS_2_ reflectance. However, there is a nonlinear modulation in the reflectance peak position shift when we apply an in-plane biaxial tensile strain. The relationship between the peak position in the reflectance spectra and the in-plane biaxial tensile strain can be described by a second-order equation:

2 $$\mathrm{Peak position}=l\left({\varepsilon }_{a}+{\varepsilon }_{b}\right)+m{\varepsilon }_{a}{\varepsilon }_{b}+n$$

where *l*, *m*, and *n* are three constants, and *ε*_*a*_ and *ε*_*b*_ are the strain components along the two lattice vectors of the MoS_2_. The first term describes the linear behavior of the peak position shift under uniaxial tensile strains along lattice vector ${\varvec{a}}$ or ${\varvec{b}}$. The second term describes the higher-order behavior in the biaxial tensile strain situation. The third term is the reflectance peak position of the unstrained MoS_2_. Since the MoS_2_ lattice vectors ${\varvec{a}}$ and ${\varvec{b}}$ are symmetrically equivalent, the tensile strains along the two directions has the same contribution factor. The fitting results show that the highest difference between the fitting curve and the first-principle-calculated peak positions is 1.76 nm, which indicates that a strain-gauge precision limit of ~ 1‰ can be obtained when the reflectance peak position is utilized to calculate the strain with the Eq. (2). Figure 3b shows the mapping image of the reflectance peak position under different in-plane biaxial tensile strains obtained from the fitted Eq. (2) (see detailed plots of reflectance in Additional file 1).

In our simulation, the lattice vector ${\varvec{a}}$ is perpendicular to the period direction of the simulated grating. Therefore, the strain ${\varepsilon }_{y}$ is equal to ${\varepsilon }_{a}$, and strain ${\varepsilon }_{x}$ equals $\sqrt{3}/2\times {\varepsilon }_{b}$. Our calculations reveal that in a MoS_2_-based grating sensor, the in-plane strain ${\varepsilon }_{x}$ can be measured by the LW edge location shift of the first-order diffraction pattern. Based on the intensity peak shift in the first-order diffraction pattern, we can utilize the second-order Eq. (2) to subtract the contribution of the in-plane strain ${\varepsilon }_{x}$ from the peak shift. Then we can quantitatively calculate the in-plane strain ${\varepsilon }_{y}$.

**Fig. 4:**
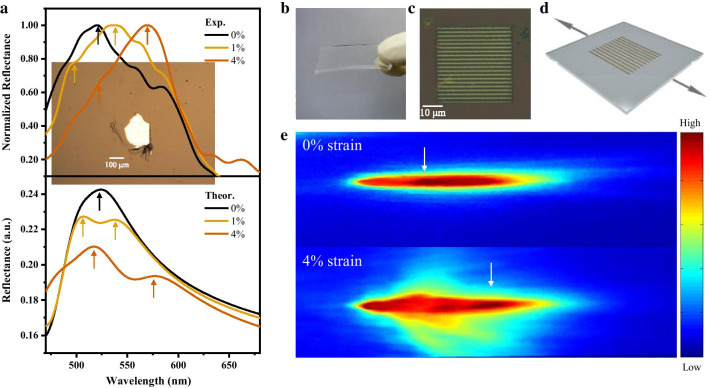
**a** Experimental results of reflectance spectra with uniaxial strains (top), and the first-principles calculated reflectance spectrum with uniaxial strains (bottom). Arrows indicate the peak locations of the reflectance. Inset, optical image of the MoS_2_ flake used for reflectance spectrum testing. **b**–**c** Optical images of the fabricated MoS_2_-based grating on PDMS. **d** Schematic diagram of MoS_2_-based grating stretched by translation stages. **e** Image of the first-order diffraction spot of the unstrained (top) and the strained (bottom) grating. White arrows indicate the intensity peak

To further experimentally study the strain-sensitivity of the MoS_2_ reflectance, we mechanically exfoliated a MoS_2_ flake (thickness of several tens of nanometers; see details in Additional file 1) and attached the flake to a flexible substrate of polydimethylsiloxane (PDMS) by a dry transfer method (shown in Fig. 4a inset). An in-plane uniaxial tensile strain was imposed on this fabricated MoS_2_ device by fixing the two sides of the substrate to two translation stages and stretching the substrate. We estimated the in-plane uniaxial tensile strain by calculating *ε*$=\delta L/L$, where $L$ is the length of the substrate between the two clips and $\delta L$ is the length change. When the strain is varied from 0 to 4%, there is a redshift of the peak position in the MoS_2_ reflectance spectrum, and the magnitude of this shift agrees well with our theoretical calculations, as shown in Fig. 4a. Figure 4b, c show the optical image of a MoS2-based reflection grating sensor with a period of 2 μm on a PDMS flexible substrate fabricated by electron-beam lithography (details in Methods). Upon stretching the PDMS substrate, an in-plane tensile strain perpendicular to the period direction is exerted on the MoS_2_-based grating device (Fig. 4d). By monitoring the intensity distribution in the first-order diffraction pattern, we observed that the intensity peak shifts further away from the center of the zero-order spot compared to the unstrained case when we introduce an in-plane tensile strain of 4% perpendicular to the period direction (Fig. 4e). No diffraction pattern location shift is obtained because the tensile strain is perpendicular to the period direction, and the spacing *d* between each strip changes little.

## Conclusion

In summary, we demonstrate a new technique for gauging in-plane biaxial strain using a MoS_2_-based reflection grating sensor. We test the concept by numerically simulating the grating with different biaxial strains up to 5%. In this new technique, the grating structure for detecting the strain component along the period direction (${\varepsilon }_{x}$) is combined with the strain-sensitivity of the MoS_2_ reflectance to act as an additional sensor to obtain the in-plane strain component perpendicular to the period direction (${\varepsilon }_{y}$). Component ${\varepsilon }_{y}$ is calculated with a second-order approximation equation and the intensity peak shift within the first-order diffraction patterns. The theoretical results are well supported by our experiments. Our work opens a path for the design of flexible grating sensors and provides a novel approach to realize one-shot in-plane biaxial strain gauges with a single grating sensor. Our approach is also applicable for other materials that have predictable reflectance response under biaxial strains and the capability to form two-dimensional single-crystal layers.

## Supplementary Information

**Additional file 1**. The supplementary information file contains a detailed description of the numerical simulation method, all results of the dielectric constants and reflectance calculated with different biaxial strains, AFM image of the MoS_2_ flake, and Raman spectrum of the MoS_2_ grating.

## Data Availability

The datasets used and analyzed during the current study are available from the corresponding author on reasonable request.

## Abbreviations

2D: Two-dimensional
VASP: Vienna Ab-initio Simulation Package
PAW: All-electron projector augmented wave
PBE: Perdew–Burke–Ernzerhof
GGA: Generalized gradient approximation
SOC: Spin–orbit coupling
BSE: Bethe–Salpeter equation
PDMS: Polydimethylsiloxane
EBL: Electron beam lithography
