# Supplementary material for: A Biaxial Strain Sensor Using a Single MoS2 Grating
Source: Nanoscale Res Lett. 2021 Feb 10;16:31. doi: 10.1186/s11671-021-03493-3 (PMC7876191; doi:10.1186/s11671-021-03493-3)
Supplement: Supplementary file 1 — Additional file 1. The supplementary information file contains a detailed description of the numerical simulation method, all results of the dielectric constants and reflectance calculated with different biaxial strains, AFM image of the MoS2 flake, and Raman spectrum of the MoS2 grating. [file 11671_2021_3493_MOESM1_ESM.docx]

**Supporting Information**

**A biaxial strain sensor using a single MoS_2_ grating**

Junxiang Xiang^#1^, Wenhui Wang^#2^, Lantian Feng^#3^, Chao Feng^1,4^, Meng Huang^1^, Ping Liu^1^, XiFeng Ren*^3^, Bin Xiang*^1^

^1^Hefei National Research Center for Physical Sciences at the Microscale, Department of Materials Science & Engineering, CAS Key Lab of Materials for Energy Conversion, University of Science and Technology of China, Hefei, Anhui 230026, China

^2^Research Laboratory for Quantum Materials, Singapore University of Technology and Design, Singapore 487372, Singapore

^3^Key Laboratory of Quantum Information, University of Science and Technology of China, Chinese Academy of Sciences, Hefei 230026, China

^4^Shandong Provincial Key Laboratory of Preparation and Measurement of Building Materials, University of Jinan, Jinan 250022, China

# These authors contributed to the work equally.

* Corresponding author: [binxiang@ustc.edu.cn](mailto:binxiang@ustc.edu.cn); [renxf@ustc.edu.cn](mailto:renxf@ustc.edu.cn)

**Note S1. First principle calculation method test**

To test our approach, we first performed optical prediction of monolayer MoS_2_. A vacuum layer of 20 Å was added to separate two MoS_2_ layers. Static energy convergence of 10^-6^ eV was used in the calculations. In the optimization step, an energy cutoff of 400eV was used, a Monkhorst-Pack k-point set of 15×15×1 was used to sample the Brillouin zone, and the geometry was relaxed until the energy converged to 10^-5^ eV. In the optical calculations, a k-point set of 6×6×1 was used as introduced by previous literature[1]. The calculated imaginary part of the transverse dielectric constant of monolayer MoS_2_ has two peaks locating at ~2.5 eV (~500 nm) and ~2.0 eV (~620nm) (Figure S1), which was consistent with previous literature [1, 2], confirming the validity of our approach.

Hybrid functional HSE06 based reflectance calculation was performed to compare with the GGA functional-based one. As shown in figure S2, the peak positions obtained from both calculations are similar. To reduce computation cost, GGA functional was chosen to predict the reflectance of the MoS_2_ flake.

**Note S2. The calculation of the MoS_2_ reflectance**

The reflectance was calculated with the equations:$Reflectivity=\frac{\left( n-1 \right)^{2}+k^{2}}{\left( n+1 \right)^{2}+k^{2}}$

$$\varepsilon_{1}=n^{2}-k^{2}$$

$$\varepsilon_{2}=2nk$$

Where n and k are the real and imaginary parts of the complex refractive index, respectively. $\varepsilon_{1}$ and $\varepsilon_{2}$ are the real and imaginary parts of the complex dielectric constant, respectively.

Systematical calculations were then performed to investigate the optical behavior of uniaxial and biaxial strained MoS_2_ flake. Both real and imaginary parts of transverse dielectric constant were predicted to have a redshift in the range of 550 nm to 600 nm (Figure S3a, and S3b) under uniaxial strain, consistent with the band structure change at K point (Figure S3c) where the direct-gap transition occurs[3]. The results of biaxial-strain results are listed in Figures S4 and S5.

Diffraction patterns were simulated with Helmholtz-Kirchhoff theorem[4]:

$$U\left( P \right)=\frac{1}{4\pi}\iint_{S} (U\frac{\partial}{\partial n}\left( \frac{1}{s} \right)-\frac{1}{s}\frac{\partial U}{\partial n})dS$$

Where *U*(*P*) is the wave function at the arbitrary point *P*, *S* is an arbitrary surface surrounding *P*, *U* is the wave function at certain area element *dS* of surface *S*, *s* is the distance between *P* and *dS*, and *n* is the normal vector of surface *S*. The simulated setup is shown in figure 1 (dimensions were shown in figure S6). The grating is illuminated by a continuous-wavelength coherent light incident at an angle of 30 degrees. The diffraction pattern is formed on the CCD parallel to the grating plane at a vertical distance of 10 cm. The grating pattern was 1µm *100 µm MoS_2_ strips separated by 2 µm gaps. We specify the coordinate origin of the image at the zero-order diffraction spot. A typical simulated diffraction image is demonstrated in figure 2a. The wavelength of 400 nm to 850 nm was included in our calculation to cover the reflectance peaks of strained and unstrained MoS_2_ flake, and to reduce unnecessary computing cost.

The coordinate system used to describe the strain in the grating device was different from the lattice coordinate of MoS_2_ (as shown in Figure S7). A convenient transformation of the two coordinates can be achieved using the following equations:

$$\varepsilon_{y}=\varepsilon_{a}$$

$$\varepsilon_{x}=\sqrt{3}/2\times\varepsilon_{b}$$

Here we omit the shape change of MoS_2_ lattice since the strain is very small.

**Note S3. MoS_2_ sample properties**

The thickness of MoS_2_ samples used in our experiments is about several tens of nanometers, assessed from the atomic force microscope (AFM) measurement of a typical fake (figure S8a). The quality of MoS_2_ grating is probed by the Raman measurement. The laser is focused on a single grating strip. The $E_{2g}^{1}$, and $A_{1g}$ Raman peaks indicate the MoS_2_ remains intact after the fabrication.

**Figures**

**Figure S1.** The real (yellow) and imaginary (blue) part of the transverse component of the dielectric constant of monolayer MoS_2_ as a function of energy.

**Figure S2.** Reflectance calculated with GGA-PBE + GW-BSE (blue), and HSE06+ GW-BSE (yellow).

**Figure S3.** The imaginary part (a) and the real part (b) of the transverse dielectric constant of MoS_2_ flake as functions of wavelength under different uniaxial strains along the lattice vector $\boldsymbol{b}$. (c) The band structure of MoS_2_ flake under different uniaxial strains along the lattice vector $\boldsymbol{b}$.

**Figure S4.** (a), (b), and (c) The real part of the transverse component of the dielectric constant of MoS_2_ flake. (d), (e), and (f) The imaginary part of the transverse component of the dielectric constant of the MoS_2_ flake. The strain is denoted as $(\varepsilon_{a}, \varepsilon_{b})$.

**Figure S5.** The reflectance of MoS_2_ flake under different in-plane strains. The strain is denoted as $(\varepsilon_{a}, \varepsilon_{b})$.

**Figure S6**. The dimensions of the simulated equipment set. The grating is illuminated by a continuous-wavelength coherent light incident at an angle of 30 degrees. The diffraction pattern is formed on the film (or CCD) parallel to the grating plane at a vertical distance of 10 cm.


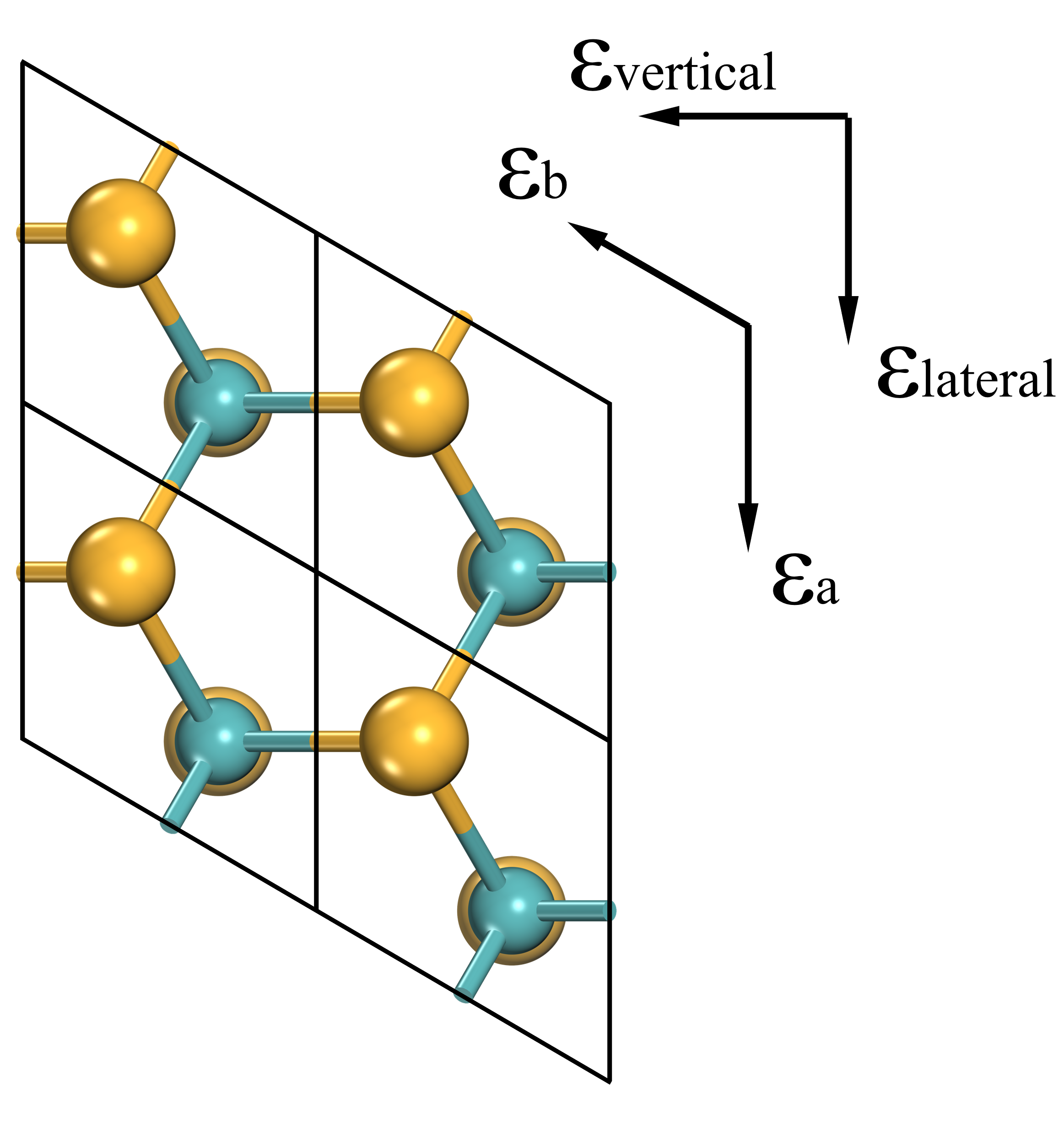


**Figure S7.** The crystal structure of MoS_2_, viewed along the z-axis, and the coordination systems used in our research.

**Figure S8.** (a) AFM image of a MoS2 flake. Inset, the line profile of the height. The thickness of the flake is about 25nm. (b) Raman spectrum, taken at one grating strip. It indicates the MoS2 remains intact after fabrication. Inset, the optical image of the MoS_2_ grating.

**References**

[1] Ramasubramaniam A (2012) Large excitonic effects in monolayers of molybdenum and tungsten dichalcogenides. Phys Rev B 86:115409

[2] Shi HL, Pan H, Zhang YW, Yakobson BI (2013) Quasiparticle band structures and optical properties of strained monolayer MoS2 and WS2. Phys Rev B 87:155304

[3] Mak KF, Lee C, Hone J, Shan J, Heinz TF (2010) Atomically Thin MoS2: A New Direct-Gap Semiconductor. Phys Rev Lett 105:136805

[4] Born M, Wolf E (1999) Principles of optics : electromagnetic theory of propagation, interference and diffraction of light 7th expanded ed. Cambridge University Press, Cambridge ; New York
